# Thinking Through Parenthood: The Role of Intolerance of Uncertainty and Personality

**DOI:** 10.1111/sjop.70107

**Published:** 2026-04-21

**Authors:** Anna Torstensson, Verona Algeroth von Thiele, Philip Millroth

**Affiliations:** ^1^ Department of Psychology Uppsala University Uppsala Sweden

**Keywords:** intolerance of uncertainty, personality, prospective parenthood, thought engagement

## Abstract

An overwhelming majority of the human population end up parenting a child, and the concept of family is arguably one of the most shared across societies. Thus, most people will at some point engage in thought processes about the prospect of becoming a parent, a prospect coupled with uncertainties (e.g., “Should I? Do I want to? Can I?”). The psychological processes underlying how individuals think about this decision—and what dispositional factors shape this deliberation—remain poorly understood. We addressed this gap by measuring thought engagement (how much individuals report thinking about parenthood and related factors) in a sample of 192 Swedish adults (58 parents, 134 non‐parents), and systematically comparing the predictive value of demographic variables, personality traits (e.g., extraversion), and dispositions toward risk and uncertainty using Bayesian multimodel inference. Intolerance of uncertainty (IU) emerged as a robust predictor of general thought engagement. Moreover, an interaction between age and occupation revealed divergent patterns: students showed increasing thought engagement with age, whereas employed individuals showed decreasing engagement. For domain‐specific thought engagement (e.g., economic concerns, parenting ability), demographic factors predominated, though personality traits also contributed, suggesting that dispositions shape what people think about rather than how much they think overall. These findings carry clinical implications: IU is a transdiagnostic vulnerability factor for anxiety disorders, and the inherently uncertain nature of the parenthood decision may be particularly distressing for some individuals. Our study provides empirical groundwork for future theoretical developments focusing on the psychological dimensions of fertility‐related decision‐making.

## Keypoints

- Intolerance of uncertainty was the clearest and most consistent predictor of overall engagement in thoughts about parenthood, indicating that uncertainty‐related dispositions may play a central role in how strongly individuals mentally engage with this life decision.
- The association between age and thought engagement depended on occupational context: among students, engagement increased with age, whereas among employed adults it decreased, pointing to distinct developmental or situational pathways in parenthood‐related deliberation.
- When specific domains of thought engagement were considered (e.g., economic situation), demographic factors were generally more predictive than broad personality traits, although dispositional characteristics still added explanatory value.

## 1 Introduction

The decision to become a parent is one of the most fundamental decisions in human life, shaping individual identities and driving societal structures. In recent decades, a global phenomenon indicates a notable decline in birth rate (Kreyenfeld 2017; Statistics Sweden 2023; United Nations 2024); with the decision to become a parent becoming an increasingly active choice, and the desire to remain childfree being more common (Athan 2020; Beets et al. 2010; Beck‐Gernsheim 2010; Morison et al. 2016). For example, in Germany, Switzerland, and Austria, over 20% of women approaching the end of their fertile age remain childfree (Kreyenfeld 2017).

Research on the topic has mainly been dominated by macro‐level perspectives. These approaches have primarily focused on aggregate patterns, often rooted in economic, sociological, and demographic factors—such as the cost–benefit calculus of childrearing, shifting gender norms, and population‐level changes in education and urbanization (e.g., Becker 1960; Balbo et al. 2013; Zaidi and Morgan 2017). Only in recent decades has micro‐level research gained traction, shifting attention to individuals by directly querying people about factors entering into the decision to become parents or not. Within this body of research, studies have identified several key factors that people, on average, report as being important for shaping the decision to become a parent or not. This includes economic factors (Beets et al. 2010), social norms (Dommermuth et al. 2011), career plans (Beets et al. 2010), the biological clock (Munson 2006), hereditary diseases (Dean and Rauscher 2017), the perceived ability to be a good parent (Holton et al. 2011), environmental concerns (Avison and Furnham 2015), and partner relationships (Rijken and Knijn 2009).

Furthermore, although little is still known about how psychological attributes of the individual may influence the process leading up to the decision, a growing amount of studies are reporting that a variety of personality traits seem associated with levels of willingness and intention to become a parent.^1^ For example, extraversion has been shown to correlate positively with a greater willingness and likelihood to have children (Avison and Furnham 2015; Berg et al. 2013; Jokela 2012), possibly because individuals with strong traits of extraversion are more social and therefore view parenthood as more rewarding (Jokela et al. 2009). Similarly, agreeableness has been shown to correlate positively with a greater willingness and likelihood to have children (Avison and Furnham 2015; Berg et al. 2013; Jokela 2012; Jokela et al. 2011), possibly because individuals who score high in agreeableness are often warm and caring toward others, which may extend to a more nurturing attitude toward children (Avison and Furnham 2015). As yet another example, conscientiousness has been shown to correlate negatively with the likelihood of becoming a parent, as individuals who score high in conscientiousness may worry that parenthood could negatively impact their careers (Avison and Furnham 2015; Jokela et al. 2011). Neuroticism has also been shown to correlate positively with ambivalence and uncertainty regarding the decision, possibly due to these individuals' difficulty in regulating emotions, leading them to focus more on the stressful aspects of parenthood—thereby complicating the decision‐making process (Jokela et al. 2011; Pinquart et al. 2008). Lastly, openness has been shown to correlate negatively with the likelihood of becoming a parent, possibly due to this trait's association with non‐traditional values and attitudes (Avison and Furnham 2015; Jokela 2012; Jokela et al. 2011).

These findings carry important implications: if stable psychological traits influence how people think about prospective parenthood, then the cognitive and emotional processes underlying this decision are not merely reactions to external circumstances. Moreover, if psychological dispositions prove to be stronger predictors than demographics, this would suggest that interventions and support—particularly for individuals experiencing distress around fertility decisions—may benefit from addressing these internal characteristics rather than focusing solely on situational factors.

However, the existing research connecting personality traits with the decision to become a parent suffers from several limitations. First, it remains unclear to what extent personality traits are predictive beyond demographic factors, and if they do, it is unclear which traits are most predictive. Second, studies have generally assessed willingness or intention, but have not addressed the extent to which people actually contemplate the decision—which, in our view, is more meaningful because intentions represent only the endpoint of a deliberative process, whereas thought engagement taps into the process itself. Two individuals may arrive at the same intention through vastly different cognitive routes: one with minimal reflection, another after extensive deliberation. Understanding this variation is essential for identifying where psychological dispositions actually operate. Importantly, these patterns have potential clinical relevance that simple measures of intention cannot capture. For example, excessive contemplation may signal distress or difficulty reaching resolution. Third, and finally, personality traits have not been compared against other psychological dispositions that may be more directly relevant. Dispositions toward risk and uncertainty^2^ are natural contenders. Not only are they correlated with personality traits (Frey et al. 2017; Millroth et al. 2020) and have proven more predictive of behavior in other contexts (Asp et al. 2026), but the decision to become a parent is, by its very nature, riddled with risk and uncertainty. Prospective parents can consult statistics on pregnancy complications, childcare costs, hereditary conditions, and even relationship outcomes—aspects that resemble calculable risks, whereas personal questions—“Will I be a good parent?”, “Will I find it fulfilling?”—remain fundamentally uncertain. It has even been theorized that parenthood itself can serve as a strategy for reducing life uncertainty, suggesting that how individuals tolerate uncertainty may be central to how they engage with this decision (Friedman et al. 1994).

### 1.1 The Present Study

The study was conducted with a Swedish sample, which provides a valuable context for several reasons. Despite having some of the most family‐friendly policies globally—including generous parental leave, subsidized childcare, and comprehensive social security—Sweden has seen a notable proportion of its population remaining childfree (approximately 13% of women and 21% of men; Statistics Sweden 2020), and in 2024 recorded its lowest birth rate in over two decades (Statistics Sweden 2025). These trends suggest that the decision to become a parent is shaped by factors beyond economic or institutional support, making Sweden an informative setting for exploring the psychological dimensions of this decision.

Our primary aim is to deepen the understanding of the psychological aspects surrounding the decision to become a parent by addressing the limitations outlined above. Whether these processes causally influence the final decision is beyond the scope of this study. However, we include exploratory analyses examining whether thought engagement patterns differ between individuals who are decided versus undecided about parenthood. Given the nascent state of research on psychological aspects of pre‐parenthood thought processes, we do not advance or test a theoretical model. Rather, our study provides empirical groundwork for future theory development by systematically identifying which categories of predictors—demographic, personality, or risk/uncertainty dispositions—warrant inclusion in such models.

We measure thought engagement, how much individuals report having thought about the decision and key factors surrounding it, both in general and across specific domains (e.g., economic conditions, partner relationships, perceived ability to be a good parent). This represents one measurable aspect of the broader thought processes involved in this decision, which may also include the content, structure, and temporal dynamics of deliberation—aspects beyond the scope of the present study.

Critically, we employ Bayesian multimodel inferences (Bergh et al. 2021; Clyde 2020; Clyde et al. 2011; Hinne et al. 2020: see Method for details), allowing us to compare the predictive value of demographic factors, personality traits, and dispositions toward risk and uncertainty within a unified framework. This approach moves beyond simply identifying links between variables to determining which predictors matter most—and whether psychological dispositions add explanatory value beyond demographics.

## 2 Method

### 2.1 Sample

The survey was distributed through social networks. Data collection yielded responses from 213 individuals, of which 192 were included in the data analysis after excluding incomplete responses (i.e., an attrition rate of 9.9%).^3^ The sample included 152 women (79%) and 40 men (21%), with participants ranging in age from 19 to 75 years (*M* = 32.3, SD = 14 years). The majority of respondents (87%) had either completed or were pursuing a university‐level education or higher. A pre‐defined sample size was not set as the analyses would be analyzed in a Bayesian framework in which the evidence would show data to be uninformative if sample size was too small. Instead, as much data as possible was collected during one semester.

Among the respondents, 58 individuals (30%) were parents (48 women and 10 men), with a mean age of 49 years and a standard deviation of approximately 11 years. The mean age for the remaining 134 respondents who were not parents at the time of the study (70%) (104 women and 30 men) was 25 years, with a standard deviation of approximately 7 years. Of the 192 respondents, 105 grew up in a major city, 61 in an urban area, and 26 in a rural area. At the time of data collection, 134 resided in a major city, 50 in an urban area, and 8 in a rural area.^4^ In terms of occupation, 80 participants were employed, 99 were students, and 13 reported “other.”

### 2.2 Procedure

Initially, participants gave written informed consent in accordance with APA guidelines for informed consent in research (American Psychological Association 2017). They were also provided the opportunity to enter a lottery for ten gift cards to a bookstore, with a total value of 1000 SEK. One hundred forty‐seven participants opted to enter the lottery.

Thereafter, respondents answered 62 items. The first 47 items were the same for parents and non‐parents, covering demographic information, measures for assessing personality traits, risk attitudes, and intolerance of uncertainty (see Measures). Respondents were then presented with items surveying the thought processes regarding the decision of becoming a parent. Here, branching was implemented to adjust item phrasing depending on whether the respondent was already a parent and thus had made the decision, or was not a parent and therefore might not have yet made the decision (see Measures).

### 2.3 Measures

#### 2.3.1 Demographic Information

The survey began with six demographic items in which respondents reported their gender, age, level of education, occupation, place of residence, and domicile. These items were included to capture patterns across demographic groups (e.g., gender, age). Interestingly, recent studies have identified a difference in fertility behavior between urban areas and rural areas, revealing a tendency for individuals in urban areas to become parents later in life (Campisi et al. 2024; Riederer and Beaujouan 2024). Campisi et al. (2024) suggested that this difference stems from greater educational and economic opportunities in urban areas, and younger women in these areas tend to postpone parenthood. Similarly, Riederer and Beaujouan (2024) found that the difference in fertility behavior diminished considerably when economic factors, gender norms, and population composition were accounted for. Additionally, statistics from Sweden and the US have shown how major cities and urban areas had a generally lower fertility rate compared to rural areas (National Center for Health Statistics 2018; Statistics Sweden 2024a, 2024b), indicating that residence and domicile may influence the decision to become a parent. To ensure that the options in these two items were not subject to individual interpretation, we included five of the largest cities in Sweden (Statistics Sweden 2024a, 2024b) in the first option, and clarified that an urban area refers to a city with more than 10,000 inhabitants in the second option.

#### 2.3.2 Personality Traits

Personality traits were assessed with a Swedish translation of the Mini‐International Personality Item Pool test (Mini‐IPIP: Donnellan et al. 2006). The Mini‐IPIP consists of 20 items, where each item presents a behavior description (e.g., “I follow a schedule”) and participants rate how accurately each statement describes them on a 5‐point rating scale. Item scores were summed to calculate a total score for five traits, onwards labeled extraversion, agreeableness, conscientiousness, neuroticism, and openness.

#### 2.3.3 Dispositions Toward Risk and Uncertainty

Building on prior research showing that dispositions toward risk and uncertainty can be meaningfully distinguished from each other when predicting real‐life behavior (Millroth and Frey 2021), we utilized the General Risk Propensity Scale (GRiPS; Zhang et al. 2019) to assess individuals' tendencies toward risk and the Short Version of the Intolerance of Uncertainty Scale (IUS‐12; Carleton et al. 2007) to measure their tendencies toward uncertainty.

The GRiPS is composed of eight items designed to evaluate a person's general inclination toward risk (e.g., “Taking risks makes life more fun”; “I commonly make risky decisions”). Participants rated each item on a scale from 1 (Strongly disagree) to 5 (Strongly agree). Scores for reversed items were summed to calculate a general risk aversion (GRA) score for each participant.

The Short Intolerance of Uncertainty Scale (Carleton et al. 2007) includes 12 items that assess an individual's general sensitivity to uncertainty (e.g., “Unforeseen events upset me greatly”; “Uncertainty keeps me from living a full life”). Seven of the items in this scale are designed to measure prospective anxiety (i.e., fear or anxiety based on future events), and five of the items are designed to measure inhibitory anxiety (i.e., uncertainty that inhibits action or experience). Responses were given on a scale from 1 (Not at all characteristic of me) to 5 (Entirely characteristic of me). The total Intolerance of Uncertainty score for each participant was obtained by summing their item scores.

#### 2.3.4 Pre‐Parenthood Thought Engagement

In the absence of existing measures capturing pre‐parental thought engagement, we developed 13 items from which we derived the outcome measures used for the inference statistics presented in the Results section (all items are available in the Supporting Information).

Not knowing beforehand whether participants could declare specific details, such as how much one is thinking about the economic conditions or merely a general propensity, we constructed both general and domain‐specific items. Five general items were answered using an 11‐point rating scale, where the respondent indicated their agreement with each statement from “Strongly *disagree*” to “*Strongly agree”*. An example of a statement in this section is “I spend a lot of time thinking about the decision to have children” for non‐parents and “I spent a lot of time thinking about the decision to have children” for parents. The design of the items in this part was based on previous research, as we identified that these statements are often used as questions in interviews and surveys regarding the decision to become a parent (Holton et al. 2011; Munson 2006; Rijken and Knijn 2009).

We subjected the five general items to Bayesian Exploratory Factor Analysis using the R‐package BayesFM (Piatek 2017) to exclude items that may have failed to tap into the general propensity that we aimed to capture (script available in the Supporting Information). This analysis showed that the posterior factor loadings for three of the items loaded on the same factor (0.686, 0.887, 0.569; and 95% HPDs of approximately ±0.150 from estimates). The remaining two items (item 2 and item 5) were dropped from further analysis, and we computed an aggregated score for each participant by summing over the three remaining items.^5^

Furthermore, respondents rated how much they think/thought about seven domain‐specific items on an 11‐point rating scale ranging from “0 = Not at all” to “10 = Very much”. Each item represented a domain that prior research has identified as commonly considered when individuals contemplate parenthood (e.g., Avison and Furnham 2015; Beets et al. 2010; Dean and Rauscher 2017; Dommermuth et al. 2011; Holton et al. 2011; Munson 2006; Rijken and Knijn 2009). The seven domains were as follows: *Economic conditions*, *Perceived ability to be a good parent*, *Biological clock*, *Hereditary diseases*, *Expectations from my surroundings (family, friends, societal norms)*, *Having a stable relationship with my partner*, and *My career*.

To assert whether thought engagement in these domains also reflected perceived importance—as an individual may, for example, consider it most essential to be a good parent, but that does not necessarily mean that they question and think about their ability to be one—respondents were presented with an item where they ranked the previously mentioned factors from 1 to 7 based on how important they were deemed in the decision to become a parent, with the most critical factor placed as number one. Bayesian estimates of Kendell's Tau showed evidence for correspondence between thought engagement and deemed importance, with correlation coefficients ranging between 0.268 and 0.477 (all BFs > 1000).

We also asked parents how planned it had been when they had children, with response options ranging from “very planned” to “very unplanned”. For non‐parents, we further asked about their desire to have children (“Yes”, “No”, “I do not know”).

### 2.4 Statistical Inferences and Reproducibility

The dataset and scripts for running the analyses are available at the OSF repository (https://osf.io/mtwfp/?view_only=1c5ad441b91b4bc996a92ea9203ca57d).

We employed two complementary Bayesian approaches: multimodel inference to identify which predictors matter most, and regression modeling to characterize the nature of specific relationships. All analyses were conducted in R.

#### 2.4.1 Bayesian Multimodel Inferences

A key challenge in exploratory research is model uncertainty: with many candidate predictors, any single model represents just one of many plausible specifications. Rather than arbitrarily selecting one model, we used Bayesian multimodel inference (Bergh et al. 2021; Clyde 2020; Clyde et al. 2011; Hinne et al. 2020) via the R‐package BAS (Clyde 2020). This approach evaluates a large space of possible models—each representing a different combination of predictors—and weights each model by its posterior probability given the data. The result is a posterior inclusion probability (PIP) for each predictor, indicating the probability (0–1) that the predictor should be included in the optimal model. PIPs above 0.50 suggest the predictor is more likely to belong in the model than not; values approaching 1.0 indicate strong evidence for inclusion.

We sampled 1000,000 models using adaptive sampling, with a uniform prior over models and the ZS‐null prior for regression coefficients (Liang et al. 2008). The ZS‐null prior is specifically designed to be conservative, penalizing model complexity and guarding against overfitting—a critical consideration given the number of candidate predictors. To assess robustness, we repeated the analyses using BIC and JZS priors; results were consistent across prior specifications (see Supporting Information). The model space included all main effects (age, gender, occupation, residence, domicile, parental status, IU, GRA, Big Five traits) and all first‐order interactions. Categorical variables (occupation, residence, domicile) were automatically dummy‐coded, with effects estimated for each level relative to a reference category.

#### 2.4.2 Bayesian Regression Modeling

To characterize the relationships identified by the multimodel inferences, we conducted follow‐up regression analyses using the R‐package brms (Bürkner 2017), which implements Bayesian generalized linear models. We used weakly informative priors, which regularize estimates without imposing strong assumptions, and report standardized coefficients (β) with 95% credible intervals (CIs). To quantify evidence for specific effects, we computed Bayes factors (BF_10_) comparing models with and without each predictor. Following standard conventions (Wetzels and Wagenmakers 2012), we interpret BF_10_ > 10 as strong evidence, BF_10_ > 30 as very strong evidence, and BF_10_ > 100 as decisive evidence for the effect. We also report Bayesian *R* ^2^ to characterize the proportion of variance explained, and compare nested models using the Leave‐One‐Out Information Criterion (LOOIC; Vehtari et al. 2017), where lower values indicate better predictive performance.

## 3 Results

### 3.1 Descriptives

**FIGURE 1:**
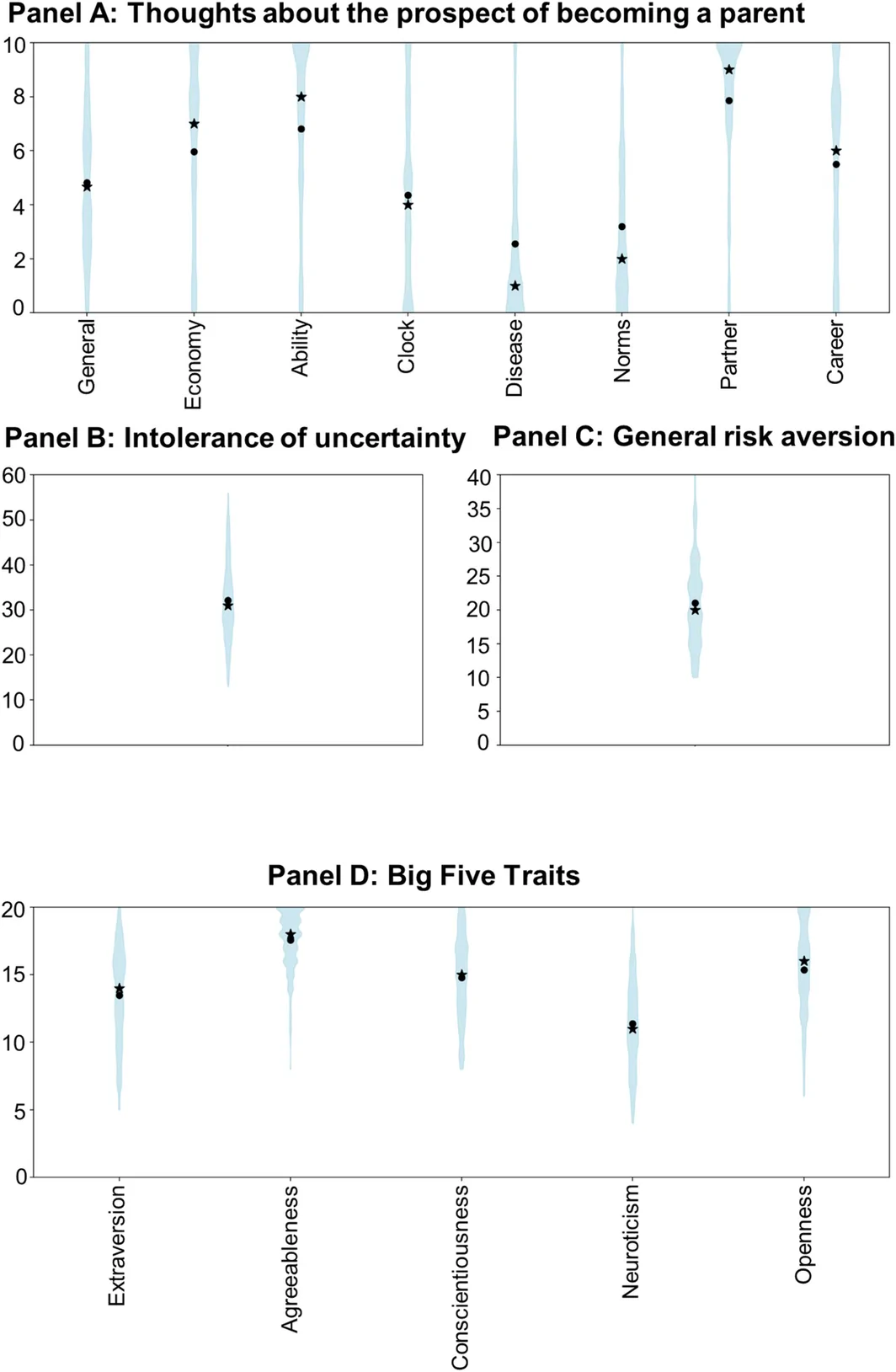
Violin plots showing the distributions of the predictors and outcome variables along with means (dots) and medians (stars).

Figure 1 provides distribution plots for the outcome measures and psychological predictors. The data show that *Partner* was the factor that participants thought most about on average, followed by *Ability*, *Economy*, and *Career*. The remaining factors (*Biological Clock*, *Heritable Disease*, and *Norms*) were generally in the lower half of the scale. The sample was, on average, relatively neutral to risk and uncertainty, but with substantial variance. The average estimates for personality were all in the upper half of the scales.

### 3.2 Estimating the Predictive Value of the Included Variables

**FIGURE 2:**
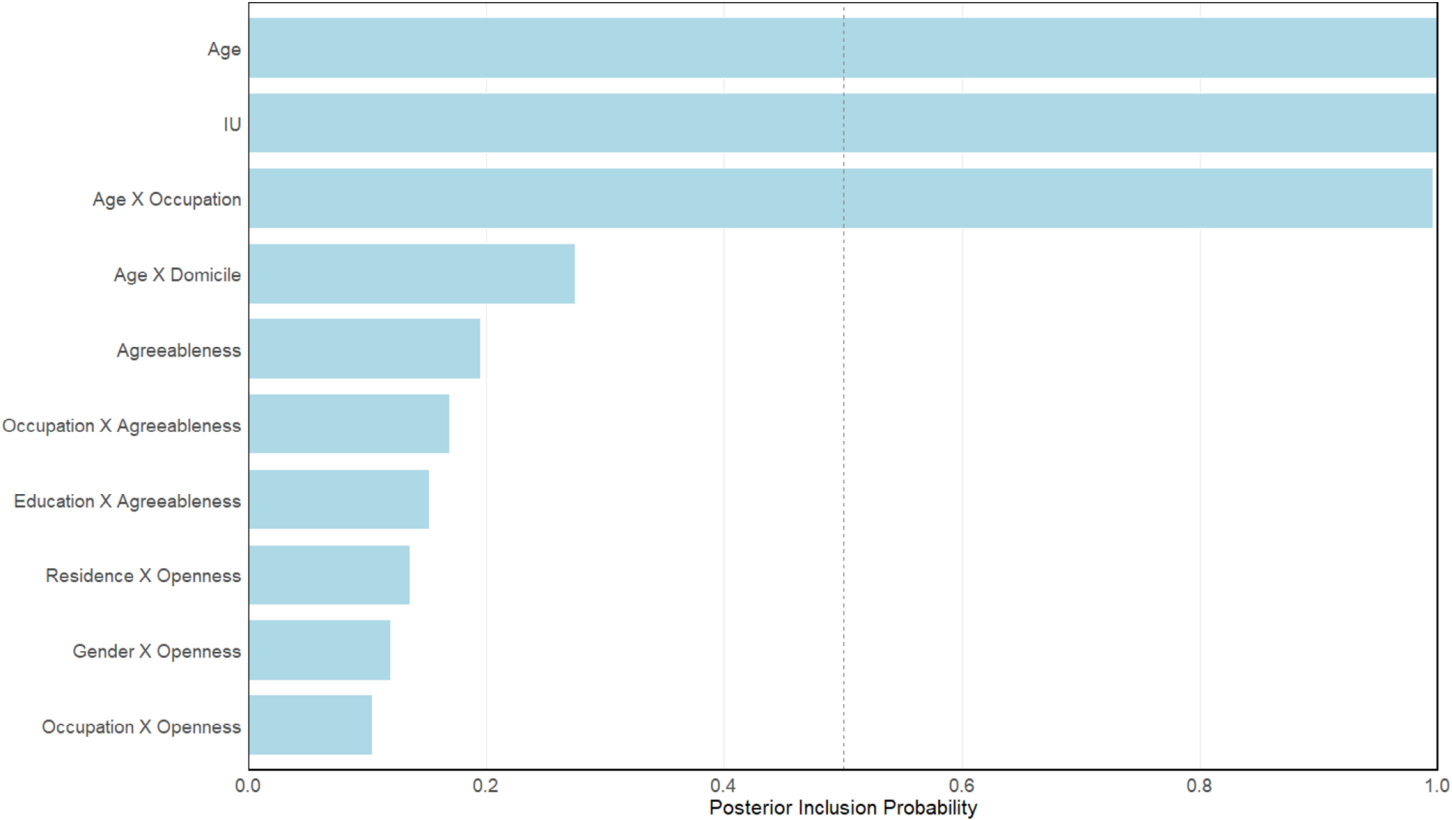
Posterior inclusion probabilities from Bayesian Adaptive Sampling (BAS) analysis predicting general thought engagement. Each bar represents the probability (0–1) that a variable should be included in the optimal predictive model.

Figure 2 shows the posterior inclusion probability for the most predictive model terms, showing that across all models, only three terms were strongly predictive of how much the individual had on *average* thought about the prospect of being a parent: age, IU, and the interaction between age and occupation. More specifically, the amount of thought given to the prospect of becoming a parent decreased with age and increased with intolerance of uncertainty (IU).

Of course, a more (or less) extensive thought engagement does not necessarily translate to the actual final decision of becoming a parent or not. With our cross‐sectional data, we cannot directly pursue a causal understanding of that relationship. However, when we ran the BAS analysis for non‐parents—predicting whether they appeared decided (i.e., reported a desire either to become parents or not) or undecided (“I don't know”) – there were no model terms with a poster inclusion probability over 0.100. This suggested that the thought engagement is not predictive of the actual decision or that our data is uninformative in this regard. This was the case also when including the domain‐specific thought engagements.

### 3.3 Unpacking the Role of Age, Education and IU in General Thought Engagement

**FIGURE 3:**
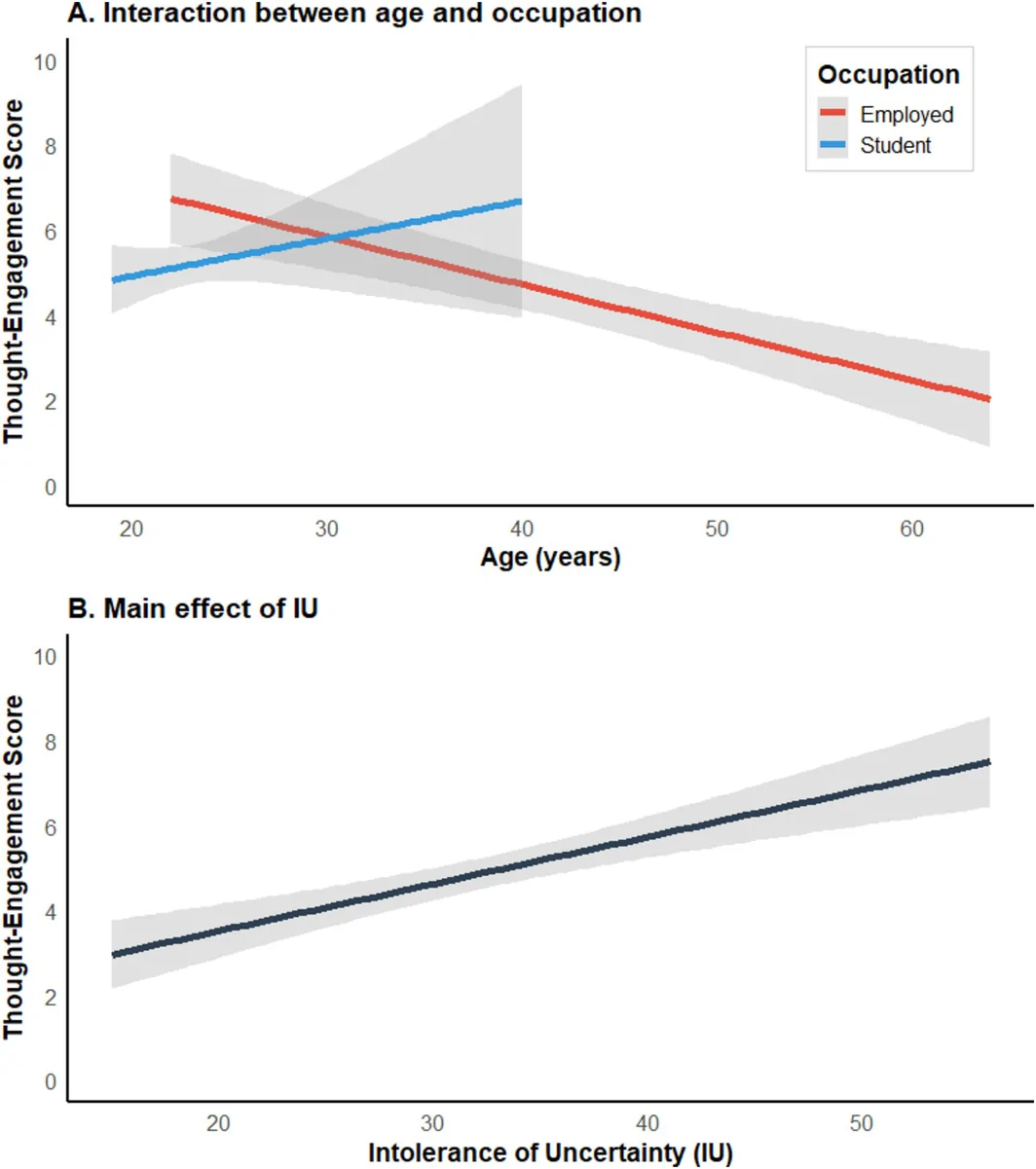
Predictors of thought engagement scores. (A) Age × occupation interaction with divergent patterns for employed versus student participants. (B) Positive main effect of intolerance of uncertainty (IU). Lines show linear fits with 95% credible intervals. Regression lines are truncated at the observed age range for each occupation group to avoid extrapolation beyond the data; students in our sample were predominantly younger (range: 19–42), whereas employed participants spanned a wider age range (range: 22–75).

To get a more detailed understanding of the relationships, we performed Bayesian generalized linear modeling using the R‐package *brms* (Bürkner 2017), with thought engagement regarding the prospect of becoming a parent as the outcome variable, and predictor terms being age, uncertainty, and the interaction between age and occupation. The results showed decisive evidence for a main effect of IU (BF_10_ = 999: *β* = 0.098, 95% CIs = 0.057; 0.139) but only anecdotally for the main effect of age (*BF*_10_ = 2.85: *β* = −0.001, 95% CIs = −0.060; 0.056). There was conclusive evidence for an interaction between age and occupation (*BF*_10_ = 1332) (Figure 3) where employed participants exhibited a negative relationship between age and thought engagement (*β* = −0.090, 95% CIs = −0.129; −0.051), whereas students exhibited a positive relationship (*β* = 0.131, 95% CIs = −0.022; 0.280). Participants in neither of the two occupation groups tended to align with the employed participants (*β* = −0.044, 95% CIs = −0.104; 0.015). The Bayesian *R* ^2^ for the model was 0.215 (95% CIs = 0.054; 0.338) compared to a model including main effects all predictors (demographics, IU, Big‐5, risk attitude), which yielded 0.271 (95% CIs = 0.056; 0.386), suggesting that little is added in explanatory power by including other psychological constructs and demographic variables. However, it also signals that there are key factors not accounted for in the present data. Because the IUS‐12 consists of two facets, inhibitory‐ and prospective anxiety we also ran two models where we replaced the total IU score with one of the facet scores. Model comparisons showed that the Leave One Out Information Criterion (LOOIC) did not differ between the model with total IU score (880) from that of prospective anxiety (880), but a model with inhibitory anxiety performed worse (894).

### 3.4 General‐ Versus Domain‐Specific Thinking

We subjected the four domain‐specific factors that participants reported thinking about the most—partner, ability, economic situation, and career—to the same Bayesian multimodel inference analyses (figures for each domain are available in the Supporting Information).

The results revealed a markedly different pattern from the general thought engagement analysis. Whereas general engagement was parsimoniously predicted by IU and the age × occupation interaction, domain‐specific engagement involved a broader and more heterogeneous set of predictors in which demographic variables and their interactions predominated, often joined by personality traits.

For the economy domain, parental status emerged as a leading predictor, and nearly all high posterior‐inclusion probability (PIP) terms were interactions involving demographic variables (e.g., age × domicile, occupation × parental status) alongside personality traits—particularly conscientiousness and agreeableness. For thought engagement about partner relationships, the strongest predictors were parental status × neuroticism, gender × age, and age as a main effect, with education × residence and age × domicile also prominent; personality entered primarily through interactions with demographic variables. The ability domain yielded the sparsest model: age was the dominant predictor, supplemented by education × residence and education × occupation, with no personality or risk/uncertainty terms exceeding the inclusion threshold. Finally, career produced the richest model, with nearly all top terms exceeding a PIP of 0.50. In addition to demographic predictors (parental status, occupation × residence), both conscientiousness and risk/uncertainty dispositions featured prominently—including GRA as a main effect, conscientiousness × GRA, education × IU, and gender × IU—making this the only domain in which risk attitudes contributed meaningfully.

Counting appearances across all four domain‐specific models, the variables show a pattern distinct from general thought engagement: residence and parental status appeared most frequently among high‐PIP terms (8 times each), followed by occupation (7 times), education and conscientiousness (6 times each), age, agreeableness, and domicile (5 times each), IU (4 times), neuroticism, GRA, and gender (3 times each), openness (2 times), and extraversion (1 time).

This pattern reinforces the central contrast: IU, the strongest psychological predictor of general engagement, receded to a secondary role at the domain level, whereas conscientiousness—absent from the general engagement model—was among the most consistent domain‐level predictors. The dissociation suggests that IU shapes the overall extent of deliberation about parenthood, whereas demographics and personality traits—conscientiousness in particular—channel attention toward specific concerns.

## 4 Discussion

Research on parenthood decisions has progressed from macro‐level analyses of economic and demographic trends to micro‐level studies identifying factors individuals report as important. Yet the psychological characteristics shaping how much—and in what ways—people think about the prospect of becoming a parent have remained largely unexamined. We addressed this research gap by asking 192 Swedish adults in a cross‐sectional survey about their thought engagement related to the decision to become a parent or not, and systematically comparing the predictive value of demographic factors, personality traits, and dispositions toward risk and uncertainty. The results revealed that intolerance of uncertainty (IU) and an interaction between age and occupation were the strongest predictors of general thought engagement, whereas domain‐specific thought engagement was frequently tied to demographic factors and, to some extent, personality traits (e.g., conscientiousness). These findings carry implications for how we conceptualize the psychological dimensions of fertility‐related decision‐making.

### 4.1 Dealing With the Inherent Risk and Uncertainty of Prospective Parenthood

The finding that IU predicted general thought engagement aligns with theoretical accounts positioning uncertainty as central to the parenthood decision. Friedman et al. (1994) argued that individuals are fundamentally motivated to reduce uncertainty and that parenthood can serve this function by providing structure and direction to life. Our findings complement this perspective by suggesting that high‐IU individuals engage more extensively with the decision—consistent with the notion that high‐IU individuals seek to gather complete information before acting (Grenier et al. 2005; Carleton 2016; Sahib et al. 2023). Importantly, this effect was specific to general thought engagement; IU did not consistently predict engagement with specific domains (e.g., economic concerns, partner relationships). This pattern suggests that high‐IU individuals do not uniformly ruminate on all aspects of the decision, but rather differ in which specific concerns capture their attention—a finding that underscores the heterogeneity of pre‐parenthood thought processes even among those who share similar dispositional profiles.

The role of IU has clinical implications; being an important transdiagnostic factor in anxiety disorders, our results imply that the prospect of becoming a parent or not could cause distress due to its inherent nature, and given its centrality in life, it likely affects many individuals at some point in their lives. That conscientiousness, which is also linked with anxiety disorders (Kotov et al. 2010), was predictive of domain‐specific engagement further points to the possibility of some people being at risk of overly ruminating about the prospect of parenthood. At its most extreme, anxiety and worry have been linked to infertility, both as outcomes of experiencing infertility as well as potential contributors to it (Newton et al. 1999; Rooney and Domar 2018; Sominsky et al. 2017).

An intriguing finding was that risk preferences did not predict thought engagement, despite being theoretically related to IU. This null finding is theoretically meaningful: it supports the Knightian distinction between risk (calculable probabilities) and uncertainty (unknowable outcomes) as psychologically distinct (Knight 1921). The decision to become a parent involves both elements, but the most personal and consequential aspects—Will I be a good parent? Will I find it fulfilling?—are fundamentally uncertain rather than risky. Our findings suggest that when facing decisions characterized by true uncertainty, it is tolerance for uncertainty rather than tolerance for risk that shapes cognitive engagement. This has implications for decision‐making research more broadly: risk and uncertainty dispositions, though correlated, may differentially predict engagement with different types of life decisions depending on whether those decisions involve calculable or incalculable outcomes.

### 4.2 The Role of Personality Traits

The lack of predictive power for personality traits in predicting general thought engagement stands in apparent contrast to previous literature linking personality to fertility intentions and outcomes (e.g., Avison and Furnham 2015; Jokela et al. 2011; Jokela 2012). However, we believe this divergence is theoretically informative rather than contradictory. Prior research has predominantly examined whether personality predicts willingness or likelihood to have children—that is, the outcome of deliberation. We examined thought engagement—the extent of deliberation itself. These are conceptually distinct: an individual high in extraversion may be more willing to have children (as prior research suggests), yet this willingness need not translate into more extensive contemplation of the decision. Indeed, if extraverts are drawn to parenthood because they anticipate social rewards (Jokela et al. 2009), they may arrive at this conclusion relatively quickly, without prolonged deliberation. Conversely, an individual high in neuroticism may be less willing to have children due to anticipated stress, yet may engage more extensively in thinking about the decision—precisely because of that ambivalence (cf. Pinquart et al. 2008).

Our finding that personality traits did predict domain‐specific thought engagement supports this interpretation. Conscientiousness and agreeableness emerged among the most frequent predictors across domains, while extraversion appeared only rarely. This pattern is consistent with the notion that certain traits steer attention toward specific concerns: conscientious individuals, who tend to be planful and concerned with long‐term consequences (Costa and McCrae 2008), may focus more on domains like career and economic stability; agreeable individuals, characterized by warmth and nurturance (Costa and McCrae 2008), may attend more to relational aspects such as partner stability and parenting ability.^6^ The relationship between personality and pre‐parenthood cognition may thus be more granular than previously recognized, with different traits channeling attention toward different concerns rather than amplifying or dampening overall deliberation.

This distinction has methodological implications for future research. Studies examining only whether personality predicts fertility outcomes may miss important psychological processes occurring earlier in the decision‐making pathway. Our findings suggest that personality shapes what people think about when contemplating parenthood, even if it does not determine how much they think about it overall.

### 4.3 Alignment With Demographic Macro‐Perspectives

Although our study adopted a micro‐level psychological approach, the findings reveal meaningful connections to the macro‐level demographic research that has traditionally dominated this field. Several demographic variables emerged as prominent predictors, especially for domain‐specific thought engagement. This pattern suggests that micro‐ and macro‐level perspectives are complementary rather than competing: the aggregate patterns observed in demographic research may, in part, reflect the cumulative effect of individual‐level psychological processes shaped by life circumstances.

The interaction between age and occupation is particularly illustrative. Macro‐level research has documented how educational attainment and employment patterns relate to fertility timing (Balbo et al. 2013), with individuals in urban areas and higher education postponing parenthood (Campisi et al. 2024). Our findings suggest a psychological mechanism that may underlie these patterns: students think more about parenthood as they age, perhaps because the decision becomes increasingly pressing as they approach the end of their educational trajectory, whereas employed individuals—having already navigated the transition to stable employment—show decreasing engagement with age. This divergence aligns with life‐course perspectives emphasizing that the timing and sequencing of major life events (education, employment, family formation) shape how individuals approach parenthood (Rijken and Knijn 2009).

Similarly, residence and domicile emerged as frequent predictors of domain‐specific thought engagement. This echoes macro‐level findings that urban–rural differences in fertility persist even after controlling for economic factors (Campisi et al. 2024; Riederer and Beaujouan 2024), suggesting that place‐based differences may operate partly through psychological channels—perhaps because urban environments expose individuals to different social norms, reference groups, or opportunity structures that shape what aspects of parenthood they contemplate (Bernardi and Klärner 2014).

These patterns are not unique to Sweden. Across post‐industrial societies, similar trends are evident: rising ages at first birth, increasing rates of voluntary childlessness, and growing diversity in family structures (Kreyenfeld 2017; Zaidi and Morgan 2017; United Nations 2024). Notably, partner relationships emerged as the domain participants reported thinking about most, consistent with research highlighting relationship context as central to fertility decision‐making (Rijken and Knijn 2009; Holton et al. 2011). As traditional pathways from marriage to parenthood become less normative (Zaidi and Morgan 2017), individuals may increasingly need to actively deliberate about whether and when their relationship context supports having children—a dynamic that our findings on thought engagement may help illuminate. While our study cannot speak directly to cross‐cultural variation, the demographic predictors that emerged—age, occupation, urbanization—are factors shaping fertility patterns globally, suggesting that the psychological processes we have identified may be relevant beyond the Swedish context.

### 4.4 Limitations

The study has several limitations. First, survey‐based approaches risk misinterpretation and reduced nuance; the decision‐making process surrounding parenthood would benefit from complementary qualitative methods. Future research employing interviews or open‐ended responses could capture the content and structure of deliberation in ways that scale‐based measures cannot. Second, our sample was a convenience sample from a single country, limiting generalizability. However, our claim is not that the specific patterns we identified will replicate across all populations. Rather, we see this study as a demonstration that psychological dispositions—particularly intolerance of uncertainty—are worthy of future investigation in this domain, and that it is possible to meaningfully assess individual differences in thought engagement. Moreover, Sweden provides an arguably informative setting for this initial investigation: its strong egalitarian norms, generous family policies, and high levels of education create a context where parenthood is largely a matter of choice rather than economic necessity or social prescription. This makes it a useful starting point for examining the psychological dimensions of the decision, though future research should examine whether these processes operate similarly in contexts with different cultural norms and institutional structures. Third, we could not determine whether any participants were part of the same couple. While this could introduce some non‐independence, we consider the impact likely to be minimal given that thought engagement is an individual‐level psychological process on which partners may diverge (Thomson 1997). Fourth, our models explained a limited proportion of variance, indicating that key factors were not captured. This likely reflects both unmeasured influences—such as relationship quality (Rijken and Knijn 2009), social network effects (Balbo and Barban 2014), and psychometric limitations of our measures. Nevertheless, the strong statistical signals that did emerge, particularly for intolerance of uncertainty, demonstrate the merits of our approach and suggest that further refinement of measurement tools could yield even clearer findings.

### 4.5 Venues for Future Research

Intriguingly, the exploratory analyses showed that none of the included variables predicted desire to have children among non‐parents, nor parents' retrospective reports of how planned their parenthood had been. Our study was not designed to examine causal effects on the actual decision, and these null findings should be interpreted cautiously given our sample size. However, if future research replicates this pattern, it would further strengthen claims that the desire to become a parent may function as a relatively universal force—culturally and/or biologically rooted—that operates independently of the situational and dispositional factors (Miller 1995), ultimately shaping how much people deliberate about the decision. This would imply a meaningful dissociation between the process of thinking about parenthood and the ultimate outcome. Several additional avenues warrant investigation. Longitudinal designs could track how thought engagement evolves across the life course—particularly across key transitions such as completing education, entering stable employment, or forming committed relationships—and whether early patterns of engagement predict later fertility outcomes. Finally, given our finding that IU predicts thought engagement, intervention research could explore whether therapeutic approaches targeting IU might benefit individuals experiencing distress around fertility decisions. Such work could bridge the gap between basic psychological research and clinical application in an area affecting most adults at some point in their lives.

## 5 Conclusion

This study sought to illuminate the psychological dimensions of a decision that typically remains examined through demographic and economic lenses. By directly measuring thought engagement and systematically comparing predictors, we identified intolerance of uncertainty (IU) and an interaction between age and occupation as central to how much individuals in general think about becoming a parent. For domain‐specific engagement, demographic factors and personality traits seemingly shaped what people think about rather than how much they think overall.

These findings point to a meaningful psychological dimension of fertility‐related decision‐making that has received insufficient attention. Intolerance of uncertainty—a transdiagnostic vulnerability factor for anxiety disorders—may render the inherently uncertain decision of whether to become a parent particularly distressing for some individuals. Given that nearly everyone contemplates parenthood at some point in life, understanding individual differences in how people engage with this decision has implications not only for clinical practice but for interpreting the fertility trends shaping societies worldwide. Our study provides empirical groundwork toward this understanding; developing a comprehensive theoretical framework for the psychology of pre‐parenthood decisions remains an important task for future research.

## Author Contributions

Anna Torstensson, Verona Algeroth von Thiele, and Philip Millroth contributed equally in conceptualization, data curation, writing – review and editing, and methodology. Anna Torstensson and Verona Algeroth von Thiele contributed equally in investigation, collected the data, project administration and writing – original draft. Philip Millroth was the PI for the project, overseeing handling of data, conducting statistical analyses, and being involved in the drafting of the manuscript; created the visualizations; involved in formal analyses, software and conducted the analyses with input from Anna Torstensson and Verona Algeroth von Thiele; and supervised and provided input foremost in the Results section.

## Funding

The authors have nothing to report.

## Disclosure

Academic Integrity Regarding AIGC: Generative AI (Claude Sonnet 4 and Opus 4.5) was used to facilitate R‐scripts as well as grammar checks.

## Ethics Statement

All procedures performed in this study involving human participants were in accordance with the ethical standards of the institutional and/or national research committee and with the 1964 Helsinki declaration and its later amendments or comparable ethical standards. The study fulfilled all necessary criteria in accordance with Swedish acts concerning the Ethical Review of Research involving humans (2003:460).

## Consent

Informed consent was obtained from all participants included in the study.

## Conflicts of Interest

The authors declare no conflicts of interest.

## Supporting information


**Figure S1:** Posterior inclusion probabilities from Bayesian Adaptive Sampling (BAS) analysis predicting thought engagement in the economy domain. Each bar represents the probability (0–1) that a variable should be included in the optimal predictive model.
**Figure S2:**. Posterior inclusion probabilities from Bayesian Adaptive Sampling (BAS) analysis predicting thought engagement in the partner domain. Each bar represents the probability (0–1) that a variable should be included in the optimal predictive model.
**Figure S3:**. Posterior inclusion probabilities from Bayesian Adaptive Sampling (BAS) analysis predicting thought engagement in the ability domain. Each bar represents the probability (0–1) that a variable should be included in the optimal predictive model.
**Figure S4:**. Posterior inclusion probabilities from Bayesian Adaptive Sampling (BAS) analysis predicting thought engagement in the career domain. Each bar represents the probability (0–1) that a variable should be included in the optimal predictive model.

## Data Availability

The data and materials in the present research were transparent. The data that support the findings of this study are available on OSF: https://osf.io/mtwfp/?view_only=1c5ad441b91b4bc996a92ea9203ca57d.

## Endnotes

^1^
We acknowledge that different traditions in personality psychology use varying terminology for these dimensions. For example, The Five Factor Model (Costa and McCrae 2008) uses “Neuroticism” whereas the Big Five/IPIP tradition uses “Emotional Stability” (Donnellan et al. 2006). Here we adopt the trait labels as used in the studies we cite when reviewing prior literature.

^2^
Traditionally, risk refers to situations with known outcome variance whereas uncertainty refers to situations with unknown probability distributions (Knight 1921; Ellsberg 1961—but see Millroth and Frey 2021, for a broadened discussion).

^3^
17 of the excluded participants stopped answering the survey when the question was posed regarding whether they were parents or not; the other four exclusions stopped answering at different time points after that question.

^4^
A major city refers to one of the five largest cities in Sweden (Statistics Sweden 2024a, 2024b), and an urban area refers to a city with more than 10,000 inhabitants.

^5^
Item 1: I spend a lot of time thinking before making the decision to have children. Item 2: I have long thought that I want to have children Item 3: I think a lot about the advantages and disadvantages of having children. Item 4: I do or will do a lot of research before making the decision to have children. Item 5: I have been uninterested in having children.

^6^
As previously noted, terminology for personality trait dimensions varies across personality traditions. In the present discussion, we interpret our findings within Costa and McCrae's Five Factor Model, as this framework structures much of the literature to which our results are compared despite the use of a measure grounded in a different tradition.
