# Supplementary material for: Thinking Through Parenthood: The Role of Intolerance of Uncertainty and Personality
Source: Scand J Psychol. 2026 Apr 21;67(5):1233–45. doi: 10.1111/sjop.70107 (PMC13546099; doi:10.1111/sjop.70107)
Supplement: Supplementary file 1 [file SJOP-67-1233-s001.docx]

**Supplementary Material for
”*Thinking Through Parenthood: The Role of Intolerance of Uncertainty and Personality”***

Figures S1-S4 summarize Posterior inclusion probabilities from Bayesian Adaptive Sampling (BAS) analysis predicting thought engagement in the economy domain (S1), partner domain (S2), ability domain (S3), and career domain (S4).

***Figure S1.*** Posterior inclusion probabilities from Bayesian Adaptive Sampling (BAS) analysis predicting thought engagement in the economy domain. Each bar represents the probability (0-1) that a variable should be included in the optimal predictive model.

***Figure S2.*** Posterior inclusion probabilities from Bayesian Adaptive Sampling (BAS) analysis predicting thought engagement in the partner domain. Each bar represents the probability (0-1) that a variable should be included in the optimal predictive model.

***Figure S3.*** Posterior inclusion probabilities from Bayesian Adaptive Sampling (BAS) analysis predicting thought engagement in the ability domain. Each bar represents the probability (0-1) that a variable should be included in the optimal predictive model.

***Figure S4.*** Posterior inclusion probabilities from Bayesian Adaptive Sampling (BAS) analysis predicting thought engagement in the career domain. Each bar represents the probability (0-1) that a variable should be included in the optimal predictive model.
